# Supplementary material for: USP7 controls NGN3 stability and pancreatic endocrine lineage development
Source: Nat Commun. 2023 Apr 28;14:2457. doi: 10.1038/s41467-023-38146-9 (PMC10147604; doi:10.1038/s41467-023-38146-9)
Supplement: Supplementary file 1 — Supplementary Information [file 41467_2023_38146_MOESM1_ESM.pdf]

SUPPLEMENTARY INFORMATION:

USP7 controls NGN3 stability and pancreatic endocrine lineage development

Teodora Manea[1#], Jessica Kristine Nelson[2,3#], Cristina Maria Garrone[1], Karin Hansson[3], Ian Evans[2,3], Axel Behrens[2,3,4,5] and Rocio Sancho[1,6,*]

[1]Centre for Gene Therapy and Regenerative Medicine, King's College London, London, UK

[2]Adult Stem Cell Laboratory, The Francis Crick Institute, 1 Midland Road, London, NW1 1AT, U.K.

[3] Cancer Stem Cell Laboratory, The Breast Cancer Now Toby Robins Research Centre, Institute of Cancer Research, 237 Fulham Road, London SW3 6JB, UK.

[4] Imperial College, Division of Cancer, Department of Surgery and Cancer, Imperial College, Exhibition Road, London SW7 2AZ, U.K.

[5] Convergence Science Centre, Imperial College, Exhibition Road, London SW7 2BU, U.K.

[6]Department of Internal Medicine III, University Hospital Carl Gustav Carus at the Technische Universität Dresden, Dresden, Germany.

# Equal contribution

* Correspondence: rocio.sancho@kcl.ac.uk

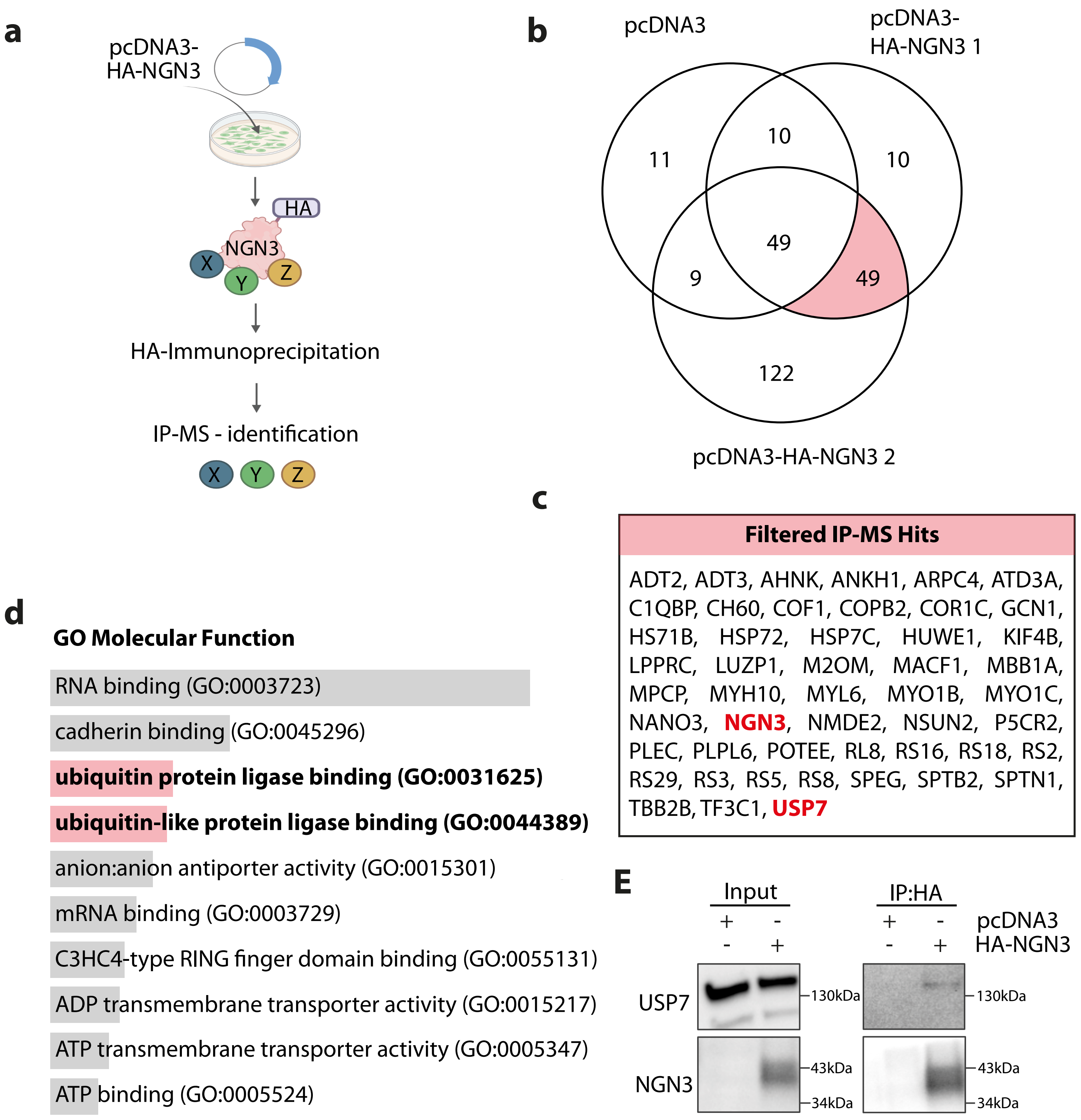

Supplementary Figure 1. IP-MS assay in HEK293A cells identifies USP7 as a possible NGN3 interactor. (a) Schematic of IP-MS strategy for NGN3 interactor identification. Created with BioRender.com. (b) Venn diagram of protein hits identified by IP-MS in the two HA-NGN3 pulldown samples and empty pcDNA3 control sample. (c) List of the 49 IP-MS hits identified in both HA-NGN3 pulldown samples but not in the pcDNA3 control sample (d) Main GO Molecular Function terms (ranked by p-value computed from the one-sided Fisher exact test) for NGN3 interactors identified through IP-MS, based on EnrichR GO Molecular Function 2021 database. Bar length indicates term significance. (e) Immunoblotting for endogenous USP7 in HA-NGN3 immunoprecipitation sample and pcDNA3 control (Image is representative of n=3 experimental replicates). Source data is available in the Source Data file.

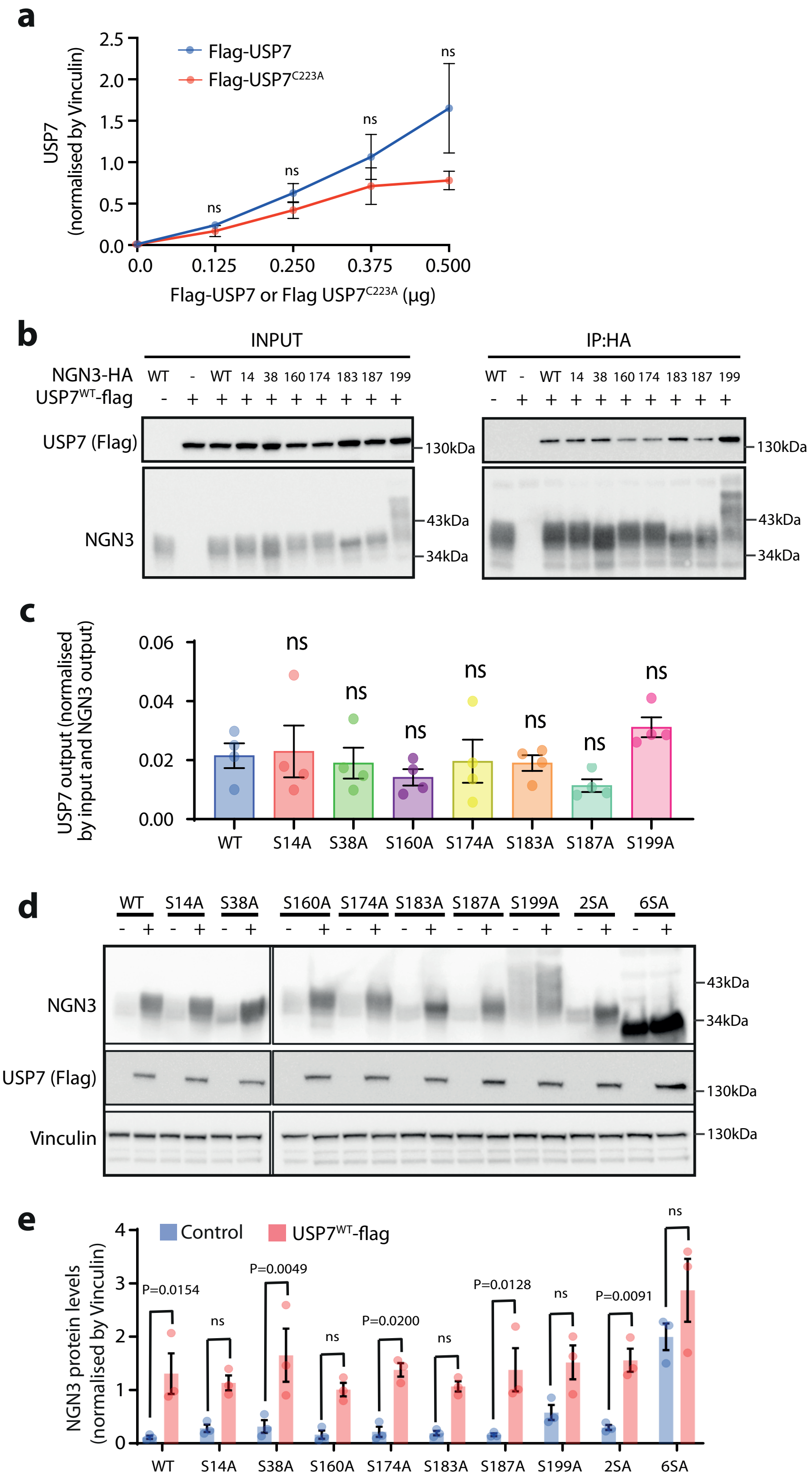

Supplementary Figure 2. USP7 interacts with NGN3 independently of NGN3 phosphorylation at previously identified phosphorylation sites. (a) Quantification of USP7 protein levels (normalized by Vinculin) in samples transfected with NGN3 and increasing concentrations of either Flag-USP7 or Flag-USP7C223A (n=3 biologically independent experiments). Plot represents mean ± SEM, and statistical significance was determined by Two-way ANOVA with Sidak's multiple comparison test, comparing each Flag-USP7C223A sample against control (Flag-USP7) sample at corresponding concentration (b) Immunoblotting for NGN3 (NGN3WT, NGN3S14A, NGN3S38A, NGN3S160A, NGN3S174A, NGN3S183A, NGN3S187A, NGN3S199A) and USP7 (Flag) in an HA-NGN3 immunoprecipitation experiment in HEK293A cells. Image is representative of n=4 biologically independent experiments. (c) Quantification of co-immunoprecipitated Flag-USP7, normalized by NGN3 output and USP7 (Flag) input from the corresponding sample. Bar graph represents mean ± SEM, and statistical significance was determined by One-way ANOVA with Dunnett's multiple comparison correction; Comparisons against NGN3WT sample (n=4 biologically independent experiments). (d) Immunoblotting for NGN3 in samples transfected with either Flag-USP7 (samples marked with "+") or the empty pcDNA3 vector control (samples marked with "-"), and NGN3 (NGN3WT, NGN3S14A, NGN3S38A, NGN3S160A, NGN3S174A, NGN3S183A, NGN3S187A or NGN3S199A). Image is representative of n=3 experiments. (e) Quantification of NGN3 (WT or mutant), normalized by Vinculin. Bar graph represents mean ± SEM, and statistical significance was determined by Two-way ANOVA with Sidak multiple comparison correction (n = 3 biologically independent experiments). Source data is available in the Source Data file.

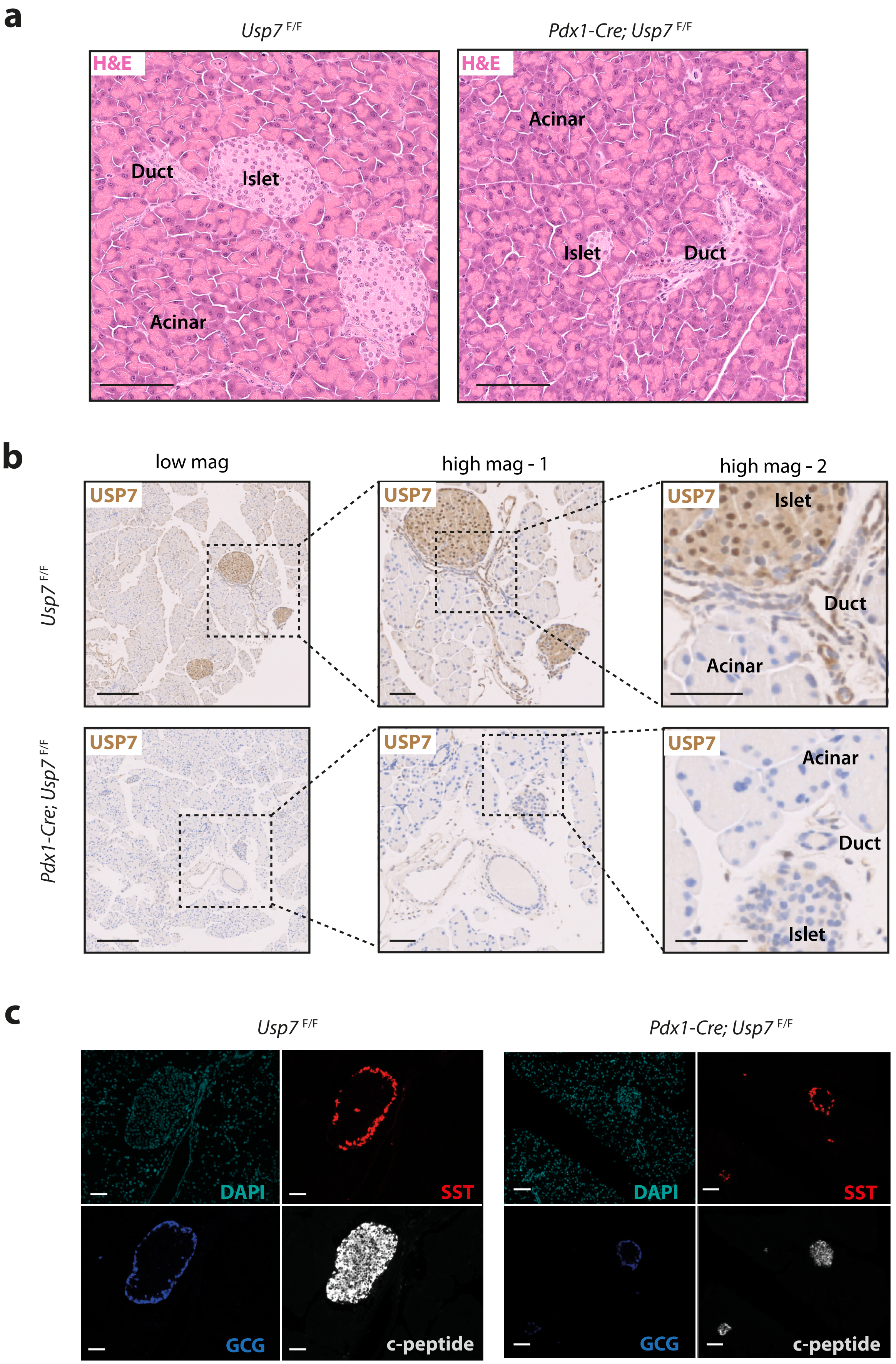

Supplementary Figure 3. Loss of USP7 in the mouse pancreas leads to reduced islet and endocrine differentiation. (a) Representative images of histological hematoxylin and eosin (H&E) analysis of Usp7 wild-type (Usp7F/F) and knock-out (Pdx1-Cre; Usp7F/F) pancreatic tissues. Scale bar is 100µm. (n=9 biologically independent animals) (b) Representative images of immunohistochemical analysis of USP7 expression in Usp7 wild-type and knockout pancreatic tissues. Scale bar is 50µm in low magnification, 200µm for high magnification-1 and -2. (n= 9 biologically independent animals) (c) Representative images of immunofluorescent staining for c-peptide (white), somatostatin (SST; red), glucagon (GCG; blue) and DAPI (teal) in Usp7 wild-type and knock-out pancreatic tissues. Scale bar is 50µm. (n= 9 biologically independent animals per group)

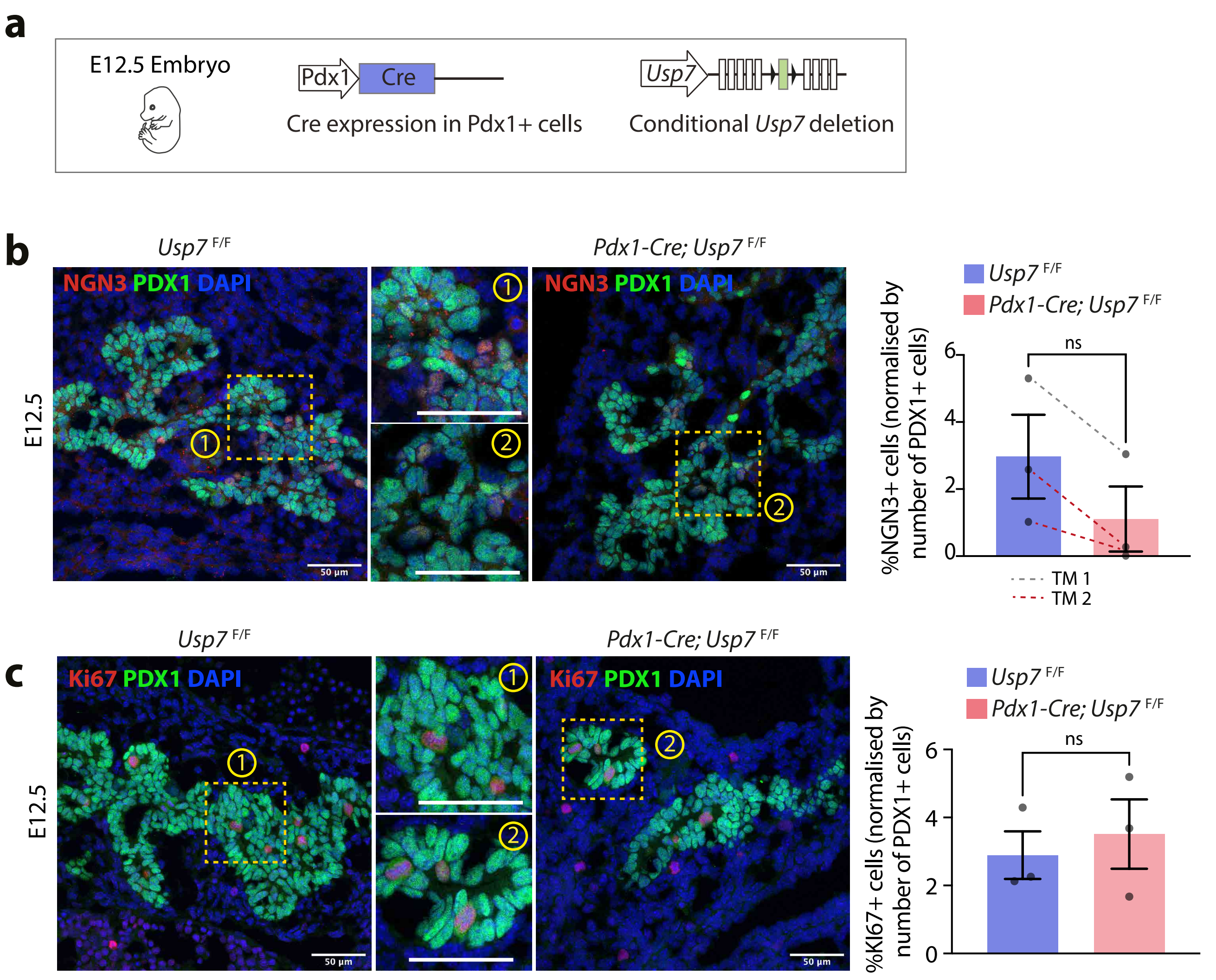

Supplementary Figure 4. Usp7 loss does not significantly impact NGN3+ progenitors or KI67+ proliferative pancreatic cells in E12.5 mouse embryos. (a) Schematic of pancreatic-specific Usp7 knock-out time mated E12.5 embryos. This diagram is adapted from Gribben et al[67] Figure 2A under Public License CC BY 4.0 (https://creativecommons.org/licenses/by/4.0/legalcode) (b) Representative images and quantification of NGN3+ endocrine progenitors (red) as a percentage of PDX1+ pancreatic cells (green) in E12.5 Usp7F/F and Pdx1-Cre; Usp7F/F embryos (n= 3 embryos per genotype with 3 frames quantified for each embryo). Plot represents mean ± SEM, and statistical significance was determined by unpaired two-tailed Student t-test. Dotted lines represent embryos from the same litter (Grey = Timed Mating 1, Red = Timed Mating 2). (c) Representative images and quantification of KI67+ proliferative pancreatic progenitors (red) as a percentage of PDX1+ pancreatic cells (green) in E12.5 Usp7F/F and Pdx1-Cre; Usp7F/F embryos (n= 3 embryos per genotype with 3 frames quantified for each embryo). Plot represents mean ± SEM, and statistical significance was determined by unpaired two-tailed Student t-test. Source data is available in the Source Data file.

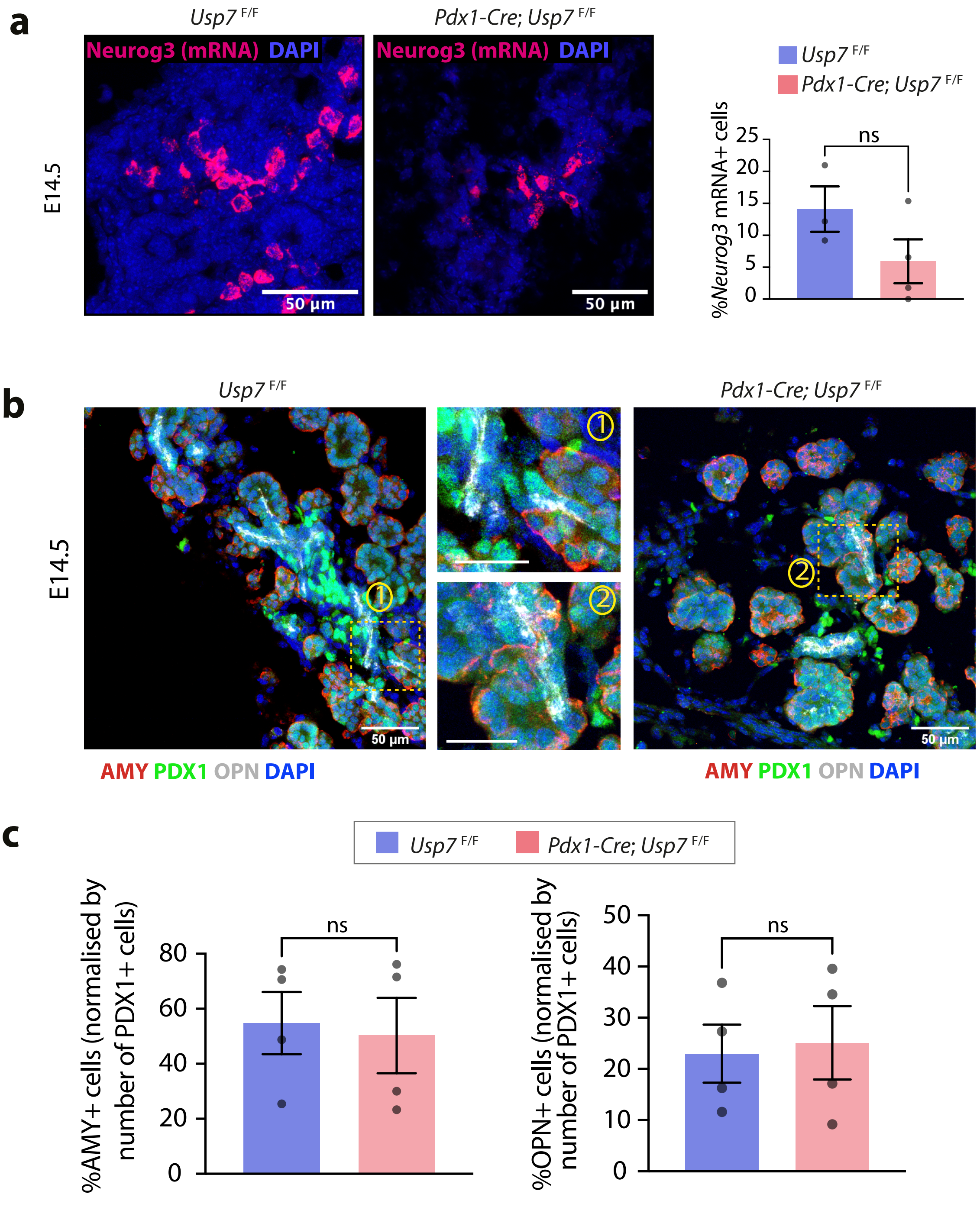

Supplementary Figure 5. USP7 loss does not significantly impact the exocrine pancreatic compartment at E14.5. (a) Representative images and quantification of Neurog3+ cells (red) as a percentage of all pancreatic cells (as determined by pancreatic morphology) in E14.5 Usp7F/F and Pdx1-Cre; Usp7F/F embryos (n= 3 Usp7F/F and 4 Pdx1-Cre; Usp7F/F biologically independent embryos with 3 frames quantified for each embryo). Plot represents mean ± SEM, and statistical significance was determined by unpaired two-tailed Student t-test. (b-c) Representative images and quantification of AMY+ (red) acinar cells and OPN+ (white) ductal cells as a percentage of PDX1+ pancreatic cells (green) in E14.5 Usp7F/F and Pdx1-Cre; Usp7F/F embryos (n= 4 embryos per genotype with 3 frames quantified for each embryo). Plot represents mean ± SEM, and statistical significance was determined by unpaired two-tailed Student t-test. Source data is available in the Source Data file.

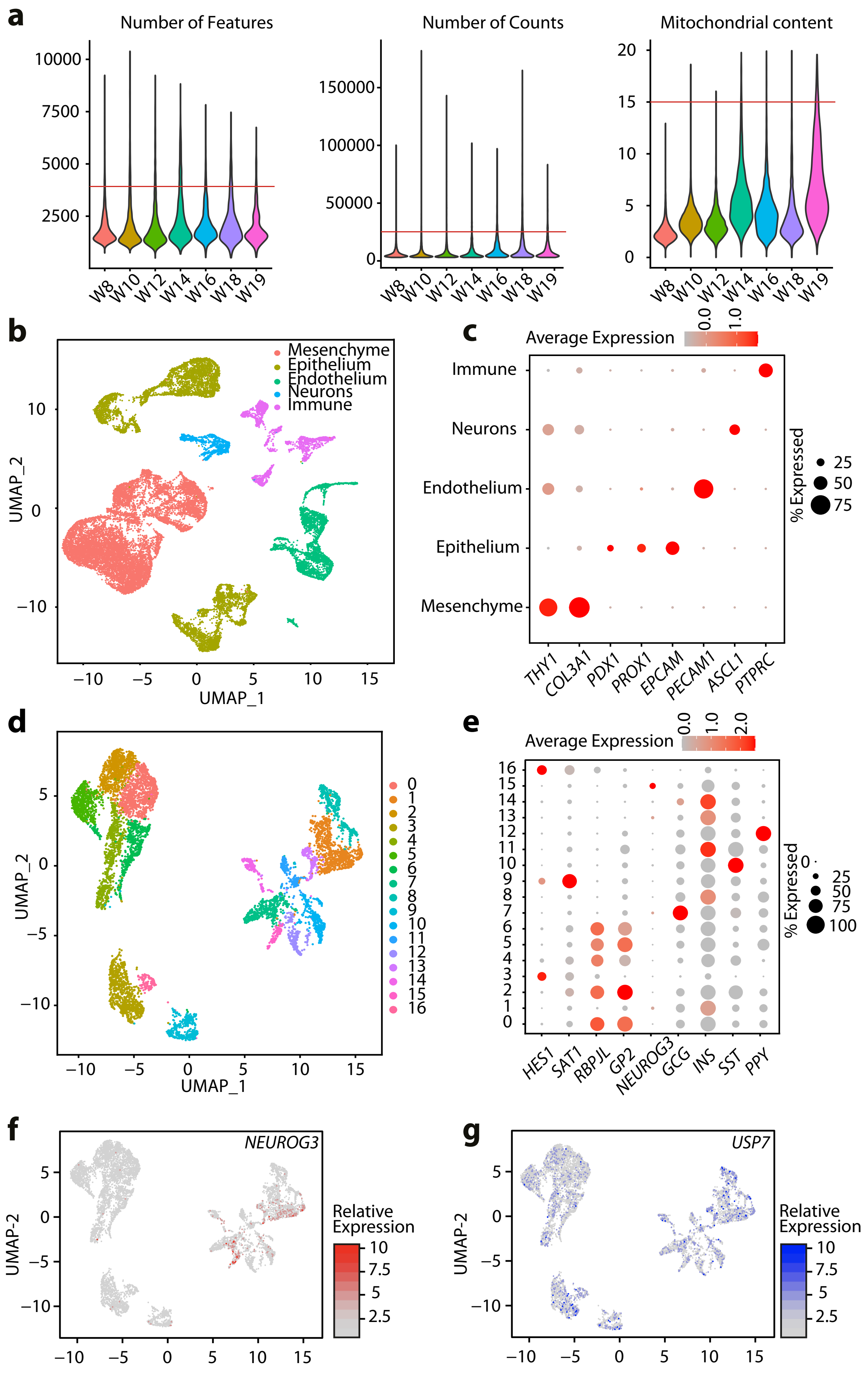

Supplementary Figure 6. Quality control and clustering of scRNA-Seq dataset. (a) Violin plots representing the number of genes detected (number of Features), the number of total RNA counts (number of Counts) and the percentage of mitochondrial genes (Mitochondrial content) present in each cell. The red line indicates the threshold selected for downstream analysis. (b) Clustering of the whole dataset transcriptome using UMAP. (c) Dotplot representing key markers selected for cell type identification. (d) Re-clustering of the epithelial cells transcriptome using UMAP. (e) DotPlot representing key markers selected for cell type identification of the epithelial cells. (f) Expression of NEUROG3 across the scRNA-seq dataset. (g) Expression of USP7 across the scRNA-seq dataset.

SUPPLEMENTARY TABLES

Supplementary Table 1 – Antibodies

ANTIBODY	Species	Company	product ID	Dilution	Application
Anti-Amylase	Goat	Santa Cruz	sc-12821	1:100	IF
Anti-Amylase	Rabbit	Sigma	A8273	1:200	IF
Anti-CK19	Rat	DSHB	TROMA-III	1:100	IF
Anti-C-peptide	Rabbit	NEB Cell signaling	4593	1:100	IF
Anti-CHGA	Rabbit	Novus	nb120-15160	1:200	IF
Anti-Glucagon	Mouse	Sigma	G2654	1:100	IF
Anti-Insulin	Guinea Pig	Dako	A0564	1:300	IF
Anti-KI67	Rabbit	Abcam	ab16667	1:200	IF
Anti-NGN3	Sheep	R&D	AF3444	1:50	IF
Anti-NKX2.2	Mouse	DSHB	74-5A5	1:200	IF
Anti-OPN	Goat	R&D	AF808	1:200	IF
Anti-PDX1	Rabbit	Cell Signalling	5679	1:200	IF
Anti-PDX1	Guinea Pig	Abcam	ab47308	1:200	IF
Anti-SST	Rat	Abcam	ab30788	1:100	IF
Anti-SST	Rabbit	Dako	A0566	1:300	IF
Anti-NGN3	Mouse	DSHB	F25A1B3	1:1000	IF/WB
Anti-USP7	Rabbit	Bethyl	A300-033A	1:500	IHC
Anti-Insulin	Mouse	Merck	K36AC10	1:2000	IHC/IF
Anti-Flag-HRP	Mouse	Sigma Aldrich	A8592	1:1000	WB
Anti-GFP	Rabbit	Cell Signalling	2956S	1:1000	WB
Anti-HA-Tag	Rabbit	Santa Cruz	sc-804	1:1000	WB
Anti-HA-Tag	Mouse	Cell Signalling	2367S	1:1000	WB
Anti-Myc-Tag	Mouse	DSHB	9e10c	1:1000	WB
Anti-USP7	Rabbit	Abcam	ab4080	1:1000	WB
Anti-Vinculin-HRP	Mouse	Santa Cruz	SC-73614-HRP	1:1000	WB
Anti-β-Catenin	Mouse	BD Transduction Laboratories	610153	1:1000	WB

Anti-goat IgG Alexa Fluor547	Donkey	Life Technology	A21447	1:200	IF
Anti-mouse IgG Alexa Fluor546	Donkey	Life Technology	A10036	1:200	IF
Anti-rabbit IgG Alexa Fluor647	Donkey	Life Technology	A31573	1:200	IF
Anti-rat IgG Alexa Fluor488	Donkey	Life Technology	A21208	1:200	IF
Anti-Guinea Pig IgG (H+L) Alexa Fluor647	Goat	Jackson ImmunoResearch	706-605-148	1:500	IF
Anti-Guinea Pig IgG (H+L) RR-X	Goat	Jackson ImmunoResearch	706-295-148	1:500	IF
Anti-Mouse IgG (H+L) Alexa Fluor488	Donkey	Jackson ImmunoResearch	715-545-151	1:500	IF
Anti-Mouse IgG (H+L) Alexa Fluor647	Donkey	Jackson ImmunoResearch	715-605-151	1:500	IF
Anti-Mouse IgG (H+L) RR-X	Donkey	Jackson ImmunoResearch	715-295-151	1:500	IF
Anti-Rabbit IgG (H+L) Alexa Fluor488	Donkey	Jackson ImmunoResearch	711-545-152	1:500	IF
Anti-Rabbit IgG (H+L) Alexa Fluor647	Donkey	Jackson ImmunoResearch	711-605-152	1:500	IF
Anti-Rabbit IgG (H+L) RR-X	Donkey	Jackson ImmunoResearch	711-295-152	1:500	IF
Anti-Rat IgG (H+L) Alexa Fluor647	Donkey	Jackson ImmunoResearch	712-605-150	1:500	IF

Anti-Sheep IgG (H+L) RR-X	Donkey	Jackson ImmunoResearch	713-295-147	1:500	IF
Anti-Mouse IgG (H+L)-HRP	Goat	Jackson ImmunoResearch	115-035-146	1:5000	WB
Anti-Rabbit IgG (H+L)-HRP	Goat	Jackson ImmunoResearch	111-035-144	1:10000	WB

Supplementary Table 2 – Primers

Primer	Sequence	Application
NGN3-S199A F	AGGTGCCCAGCGCCCCATCC	Cloning
NGN3-S199A R	GGATGGGGCGCTGGGCACCT	Cloning
NGN3-S174A F	GCTCTATCTACGCCCCAGTCTCCCAA	Cloning
NGN3-S174A R	TTGGGAGACTGGGGCGTAGATAGAGC	Cloning
NGN3-S160A F	GAGCTGGGGGCCCCCGGAGG	Cloning
NGN3-S160A R	CCTCCGGGGGCCCCCAGCTC	Cloning
NGN3-S38A F	CCCCACCTGCCCCCACTCTC	Cloning
NGN3-S38A R	GAGAGTGGGGGCAGGTGGGG	Cloning
NGN3-S14A F	CCATCCAAGTGGCCCCAGAGACACAAC	Cloning
NGN3-S14A R	GTTGTGTCTCTGGGGCCACTTGGATGG	Cloning
USP7-C223A F	AGGGAGCGACTGCTTACATGAACAG	Cloning
USP7-C223A R	GTCCCTCGCTGACGAATGTACTTGT	Cloning
GAPDH F	TTGCTTGTAGCCAAATTCGTTG	RT-qPCR
GAPDH R	ATTGCCCTCAACGACCACTTT	RT-qPCR
GCG F	TTCCCAGAAGAGGTCGCCATTGTT	RT-qPCR
GCG R	CAACCAGTTTATAAAGTCCCTGGCGG	RT-qPCR
INS F	AGGCTTCTTCTACACACCCAAG	RT-qPCR
INS R	CACAATGCCACGCTTCTG	RT-qPCR
NGN3 F	CTCGGACCCCATTCTCTCTT	RT-qPCR
NGN3 R	CTTCTGGTCGCCAAGTTCA	RT-qPCR
SST F	GAGAATGATGCCCTGGAACCTGAAGA	RT-qPCR
SST R	ATTCTTGCAGCCAGCTTTGCGT	RT-qPCR
