# Supplementary material for: USP7 controls NGN3 stability and pancreatic endocrine lineage development
Source: Nat Commun. 2023 Apr 28;14:2457. doi: 10.1038/s41467-023-38146-9 (PMC10147604; doi:10.1038/s41467-023-38146-9)
Supplement: Supplementary file 3 — Reporting Summary [file 41467_2023_38146_MOESM3_ESM.pdf]

nature portfolio

Corresponding author(s): Rocio Sancho

Last updated by author(s): 29 March 2023

Reporting Summary

Nature Portfolio wishes to improve the reproducibility of the work that we publish. This form provides structure for consistency and transparency in reporting. For further information on Nature Portfolio policies, see our Editorial Policies and the Editorial Policy Checklist.

Statistics

For all statistical analyses, confirm that the following items are present in the figure legend, table legend, main text, or Methods section.

n/a	Confirmed	
☐	☒	The exact sample size (*n*) for each experimental group/condition, given as a discrete number and unit of measurement
☐	☒	A statement on whether measurements were taken from distinct samples or whether the same sample was measured repeatedly
☐	☒	The statistical test(s) used AND whether they are one- or two-sided<br>*Only common tests should be described solely by name; describe more complex techniques in the Methods section.*
☐	☒	A description of all covariates tested
☐	☒	A description of any assumptions or corrections, such as tests of normality and adjustment for multiple comparisons
☐	☒	A full description of the statistical parameters including central tendency (e.g. means) or other basic estimates (e.g. regression coefficient) AND variation (e.g. standard deviation) or associated estimates of uncertainty (e.g. confidence intervals)
☐	☒	For null hypothesis testing, the test statistic (e.g. *F*, *t*, *r*) with confidence intervals, effect sizes, degrees of freedom and *P* value noted<br>*Give P values as exact values whenever suitable.*
☒	☐	For Bayesian analysis, information on the choice of priors and Markov chain Monte Carlo settings
☒	☐	For hierarchical and complex designs, identification of the appropriate level for tests and full reporting of outcomes
☒	☐	Estimates of effect sizes (e.g. Cohen's *d*, Pearson's *r*), indicating how they were calculated

Our web collection on statistics for biologists contains articles on many of the points above.

Software and code

Policy information about availability of computer code

Data collection	Leica Application Suite X (LAS X) was used to adquire confocal images, Zeiss Axio Scan was used to adquire tissue section images.
Data analysis	Data was analysed using the following softwares: GraphPad Prism (v.9), Image J (v1.53), Zen 3.0 software package, Proteome Discoverer (ThermoScientific; v2.5), Xcalibur (v4.3), Scaffold (v5.1.0). Custom code used for the analysis of NGS data was written in R (v4.1.1) using the Seurat package (v 4.0.4) and is available from Zenodo (DOI 10.5281/zenodo.6525040).

For manuscripts utilizing custom algorithms or software that are central to the research but not yet described in published literature, software must be made available to editors and reviewers. We strongly encourage code deposition in a community repository (e.g. GitHub). See the Nature Portfolio guidelines for submitting code & software for further information.

Data

Policy information about availability of data

All manuscripts must include a data availability statement. This statement should provide the following information, where applicable:

- Accession codes, unique identifiers, or web links for publicly available datasets
- A description of any restrictions on data availability
- For clinical datasets or third party data, please ensure that the statement adheres to our policy

DATA AVAILABILITY: The mass spectrometry proteomics data shown in Supplementary Fig. 1 are available via ProteomeXchange with identifier PXD033691. Additionally, the publicly available dataset used for Fig. 5 has been obtained and used in this manuscript (OMIX23638). Data will be available after publication.

CODE AVAILABILITY: Custom code used for the analysis of NGS data was written in R and is available from from GitHub (https://github.com/SanchoLab/scRNAseq_Human_Foetal_Pancreas). Data will be available after publication.

Human research participants

Policy information about studies involving human research participants and Sex and Gender in Research.

Reporting on sex and gender	No human research participants were used in this study.
Population characteristics	No human research participants were used in this study.
Recruitment	No human research participants were used in this study.
Ethics oversight	No human research participants were used in this study.

Note that full information on the approval of the study protocol must also be provided in the manuscript.

Field-specific reporting

Please select the one below that is the best fit for your research. If you are not sure, read the appropriate sections before making your selection.

☒ Life sciences ☐ Behavioural & social sciences ☐ Ecological, evolutionary & environmental sciences

For a reference copy of the document with all sections, see nature.com/documents/nr-reporting-summary-flat.pdf

Life sciences study design

All studies must disclose on these points even when the disclosure is negative.

Sample size	The exact sample sizes (n) used to calculate statistics are provided in the figure legends. No statistical methods were used to predetermine sample size. Sample size was determined to be adequate based on the magnitude and consistency of measurable differences between groups based on our and others previous studies (DOI: 10.7554/eLife.71596; DOI: 10.1038/s41586-022-04888-7; DOI: 10.1038/s41467-022-29684-9).
Data exclusions	No data was excluded from the analysis.
Replication	All experiments were repeated with similar results a minimum of three times, unless otherwise specified. All immunoblots and micrograph images are representative of a minimum of three biologically independent experiments.
Randomization	Adult mice were randomly assigned for the experiments (weight and glucose measurements). There was no allocation of test subjects groups for any other experiments, thus randomization is not relevant beyond animal experiments in this study.
Blinding	In vivo experiments and adult mice image analysis were perform blinded as the genotype was not known by the investigators until the end of the analysis. Embryo analysis was not performed blinded as the investigators new the genotype of the embryos. No blinding was applied to in vitro experiments analysis as each investigator performing a given experiment labeled the corresponding samples and performed the analysis

Reporting for specific materials, systems and methods

We require information from authors about some types of materials, experimental systems and methods used in many studies. Here, indicate whether each material, system or method listed is relevant to your study. If you are not sure if a list item applies to your research, read the appropriate section before selecting a response.

Materials & experimental systems

n/a	Involved in the study	
☐	☒	Antibodies
☐	☒	Eukaryotic cell lines
☒	☐	Palaeontology and archaeology
☐	☒	Animals and other organisms
☒	☐	Clinical data
☒	☐	Dual use research of concern

Methods

n/a	Involved in the study	
☒	☐	ChIP-seq
☒	☐	Flow cytometry
☒	☐	MRI-based neuroimaging

Antibodies

Antibodies used

Anti-Amylase Goat Santa Cruz sc-12821
Anti-Amylase Rabbit Sigma A8273
Anti-CK19 Rat DSHB TROMA-III
Anti-C-peptide Rabbit NEB Cell signaling 4593
Anti-CHGA Rabbit Novus nb120-15160
Anti-Glucagon Mouse Sigma G2654
Anti-Insulin Guinea Pig Dako A0564
Anti-KI67 Rabbit Abcam ab16667
Anti-NGN3 Sheep R&D AF3444
Anti-NKX2.2 Mouse DSHB 74-5A5
Anti-OPN Goat R&D AF808
Anti-PDX1 Rabbit Cell Signalling 5679
Anti-PDX1 Guinea Pig Abcam ab47308
Anti-Somatostatin Rat Abcam ab30788
Anti-Somatostatin Rabbit Dako A0566
Anti-NGN3 Mouse DSHB F25A1B3
Anti-USP7 Rabbit Bethyl A300-033A
Anti-Insulin Mouse Merck K36AC10
Anti-Flag-HRP Mouse Sigma Aldrich A8592
Anti-GFP Rabbit Cell Signalling 2956S
Anti-HA-Tag Rabbit Santa Cruz sc-804
Anti-HA-Tag Mouse Cell Signalling 2367S
Anti-Myc-Tag Mouse DSHB 9e10c
Anti-USP7 Rabbit Abcam ab4080
Anti-Vinculin-HRP Mouse Santa Cruz SC-73614-HRP
Anti-β-Catenin Mouse BD Transduction Laboratories 610153
Anti-goat IgG Alexa Fluor547 Donkey Life Technology A21447
Anti-mouse IgG Alexa Fluor546 Donkey Life Technology A10036
Anti-rabbit IgG Alexa Fluor647 Donkey Life Technology A31573
Anti-rat IgG Alexa Fluor488 Donkey Life Technology A21208
Anti-Guinea Pig IgG (H+L) Alexa Fluor647 Goat Jackson ImmunoResearch 706-605-148
Anti-Guinea Pig IgG (H+L) RR-X Goat Jackson ImmunoResearch 706-295-148
Anti-Mouse IgG (H+L) Alexa Fluor488 Donkey Jackson ImmunoResearch 715-545-151
Anti-Mouse IgG (H+L) Alexa Fluor647 Donkey Jackson ImmunoResearch 715-605-151
Anti-Mouse IgG (H+L) RR-X Donkey Jackson ImmunoResearch 715-295-151
Anti-Rabbit IgG (H+L) Alexa Fluor488 Donkey Jackson ImmunoResearch 711-545-152
Anti-Rabbit IgG (H+L) Alexa Fluor647 Donkey Jackson ImmunoResearch 711-605-152
Anti-Rabbit IgG (H+L) RR-X Donkey Jackson ImmunoResearch 711-295-152
Anti-Rat IgG (H+L) Alexa Fluor647 Donkey Jackson ImmunoResearch 712-605-150
Anti-Sheep IgG (H+L) RR-X Donkey Jackson ImmunoResearch 713-295-147
Anti-Mouse IgG (H+L)-HRP Goat Jackson ImmunoResearch 115-035-146
Anti-Rabbit IgG (H+L)-HRP Goat Jackson ImmunoResearch 111-035-144

Validation

All antibodies' validation statements for the species and application can be found on the manufacturer's website upon search of specific catalog numbers specified above for each of the antibodies.

Eukaryotic cell lines

Policy information about cell lines and Sex and Gender in Research

Cell line source(s)

Human Embryonic Kindney 293A cells were obtained from the Cell Service Facility at the Francis Crick Institute. Kute4 (iPSCs) were obtained from hiPSCi.

Authentication

293A were authenticated by the vendor (Thermo Fisher) prior to their transfer to the Francis Crick cell service facility. Kute4 were fully characterized by hiPSCi (https://hpscreg.eu/docs/uploads//certificate_of_analysis/d5a19a19f2dc43f2b394d62218ef86b6.pdf). Cells grew, performed and showed morphology as expected. No additional specific authentication was performed.

Mycoplasma contamination

Cells were negative for mycoplasma contamination as per our centre screening using PCR-based analysis for mycoplasma.

Commonly misidentified lines
(See ICLAC register)

No commonly missidentified cell lines were used in this study

Animals and other research organisms

Policy information about studies involving animals; ARRIVE guidelines recommended for reporting animal research, and Sex and Gender in Research

Laboratory animals	This study includes adult mice (5-8 weeks) and mouse embryos (E12.5, E14.5). The strains used were USP7flox (Kon et al. 2011) and Pdxl-Cre (Hingorani et al. 2003). These lines were inter-crossed to generate the desired genotypes on a C57BL/6 background.
Wild animals	No wild animals were used in this study.
Reporting on sex	The mice used in this study were not disaggregated by sex, and both males and females were used in this study. The phenotype and mechanism described in this study is not sex-dependent.
Field-collected samples	The study did not include samples collected from the field.
Ethics oversight	All animal experiments were approved by the Francis Crick Institute and Institute of Cancer Research Animal Ethics Committees and conformed to UK Home Office regulations under the Animals (Scientific Procedures) Act 1986 including Amendment Regulations 2012.

Note that full information on the approval of the study protocol must also be provided in the manuscript.
